# Supplementary material for: The Properties of Activated Carbons Functionalized with an Antibacterial Agent and a New SufA Protease Inhibitor
Source: Materials (Basel). 2023 Feb 1;16(3):1263. doi: 10.3390/ma16031263 (PMC9920905; doi:10.3390/ma16031263)
Supplement: Supplementary file 1 [file materials-16-01263-s001.zip › materials-2138263-supplementary.pdf]

materials

Supplementary Materials

The Properties of Activated Carbons Functionalized with an Antibacterial Agent and a New SufA Protease Inhibitor

Ewa Burchacka [1,*], Katarzyna Pstrowska [1], Michał Bryk [1], Filip Maciejowski [1], Marek Kułażyński [2] and Katarzyna Chojnacka [1]

[1] Department of Chemistry, Wroclaw University of Science and Technology, Wyspiańskiego Str. 27, 50-370 Wroclaw, Poland
[2] Innovation and Implementation Company Ekomotor Ltd., 1A Wyścigowa Street, 53-011 Wroclaw, Poland
* Correspondence: ewa.burchacka@pwr.edu.pl

Table of Contents

Figure S1 The standard curve for the gentamicin (G) - ninhydrin complex
Figure S2 The standard curve for the Cbz-Ile-Ser-6-AmNpth^P(OPh)$_2$ (**11**)
Figure S3 ^{1}H NMR for the 6-(Methoxycarbonyl)-2-naphthoic acid (**2**)
Figure S4 ^{1}H NMR for the Methyl 6-carbamoyl-2-naphthoate (**3**)
Figure S5 ^{1}H NMR for the Methyl 6-cyano-2-naphthoate (**4**)
Figure S6 ^{1}H NMR for the 6-(Hydroxymethyl)-2-naphthonitrile (**5**)
Figure S7 ^{1}H NMR for the Cbz-6-CN-NphthP(OPh)$_2$ (**7**)
Figure S8 ^{31}P NMR for the Cbz-6-CN-NphthP(OPh)$_2$ (**7**)
Figure S9 ^{1}H NMR for the Cbz-6-AmNphth^P(OPh)$_2$ (**8**)
Figure S10 ^{31}P NMR for the Cbz-6-AmNphth^P(OPh)$_2$ (**8**)
Figure S11 MS analysis for Cbz-Ile-Ser-6-AmNphth^P(OPh)$_2$ (**11**)
Figure S12 ^{1}H and ^{31}P NMR for the Cbz-Ile-Ser-6-AmNphth^P(OPh)$_2$ (**11**)
Figure S13 ^{31}P NMR for the Cbz-Ile-Ser-6-AmNphth^P(OPh)$_2$ (**11**)

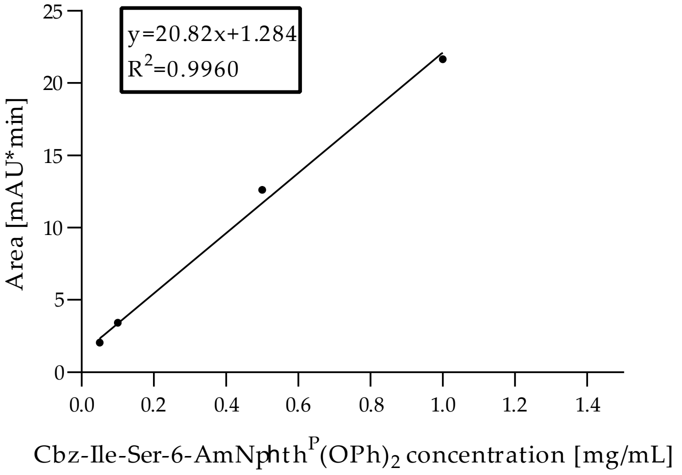

Figure S1. The standard curve for the gentamicin (Gt)-ninhydrin complex.

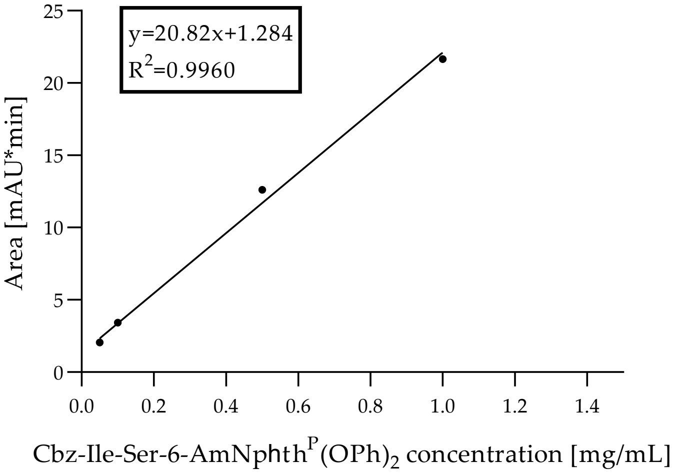

Figure S2. The standard curve for the Cbz-Ile-Ser-6-AmNphth^P(OPh)$_2$ (**11**).

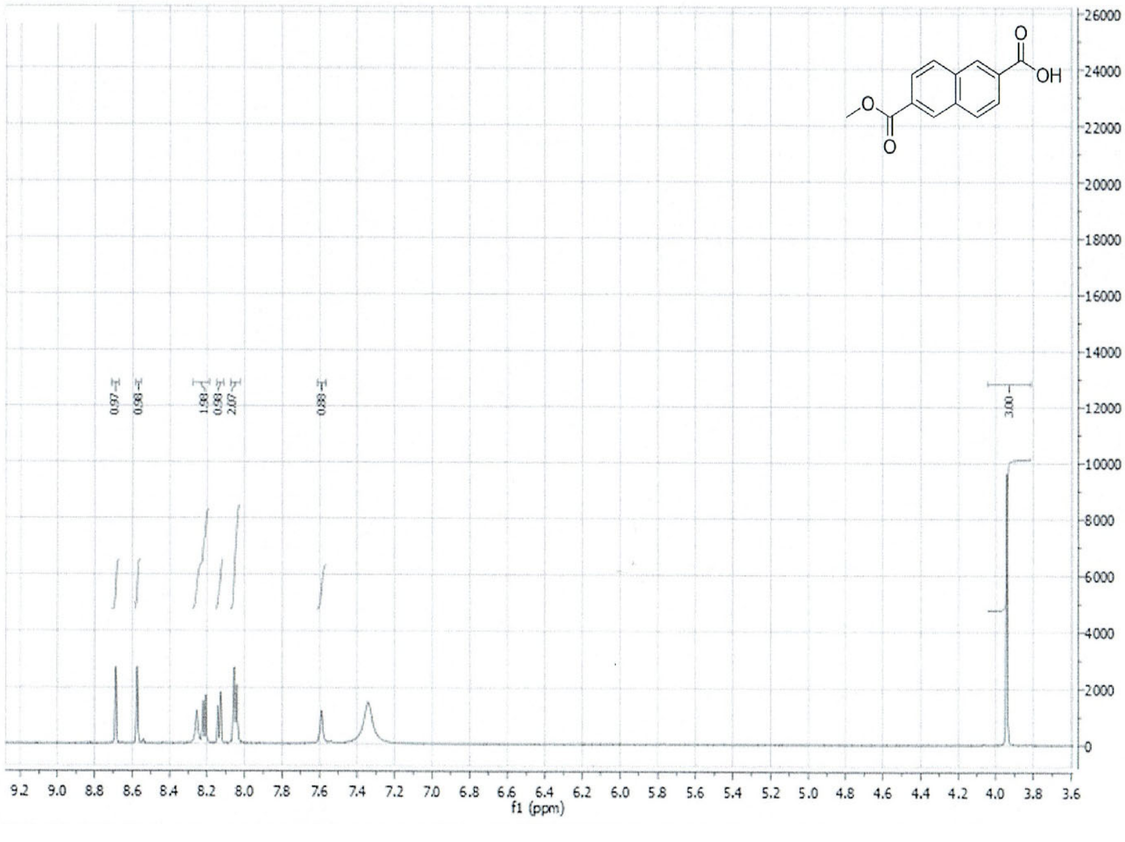

Figure S3. ^{1}H NMR for the 6-(Methoxycarbonyl)-2-naphthoic acid (**2**).

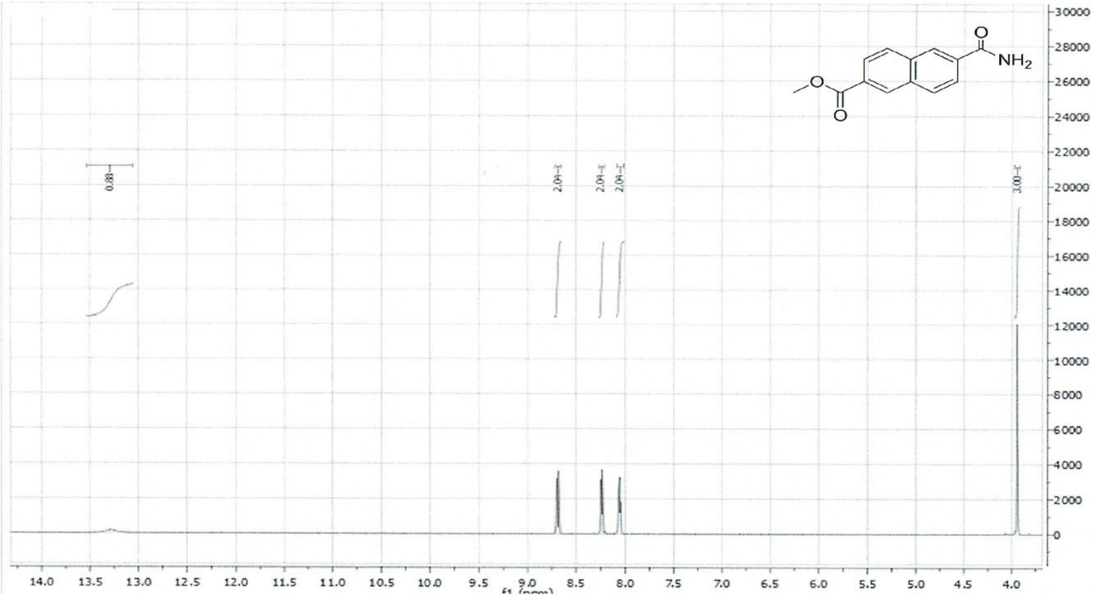

Figure S4. ^{1}H NMR for the Methyl 6-carbamoyl-2-naphthoate (**3**).

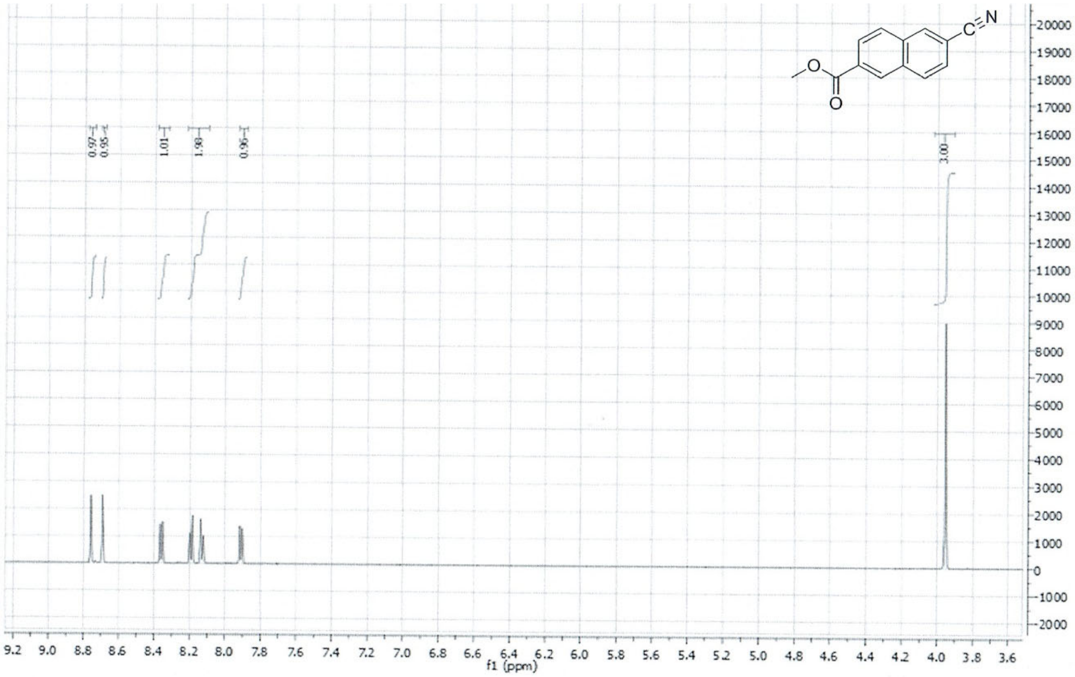

Figure S5. ^{1}H NMR for the Methyl 6-cyano-2-naphthoate (**4**).

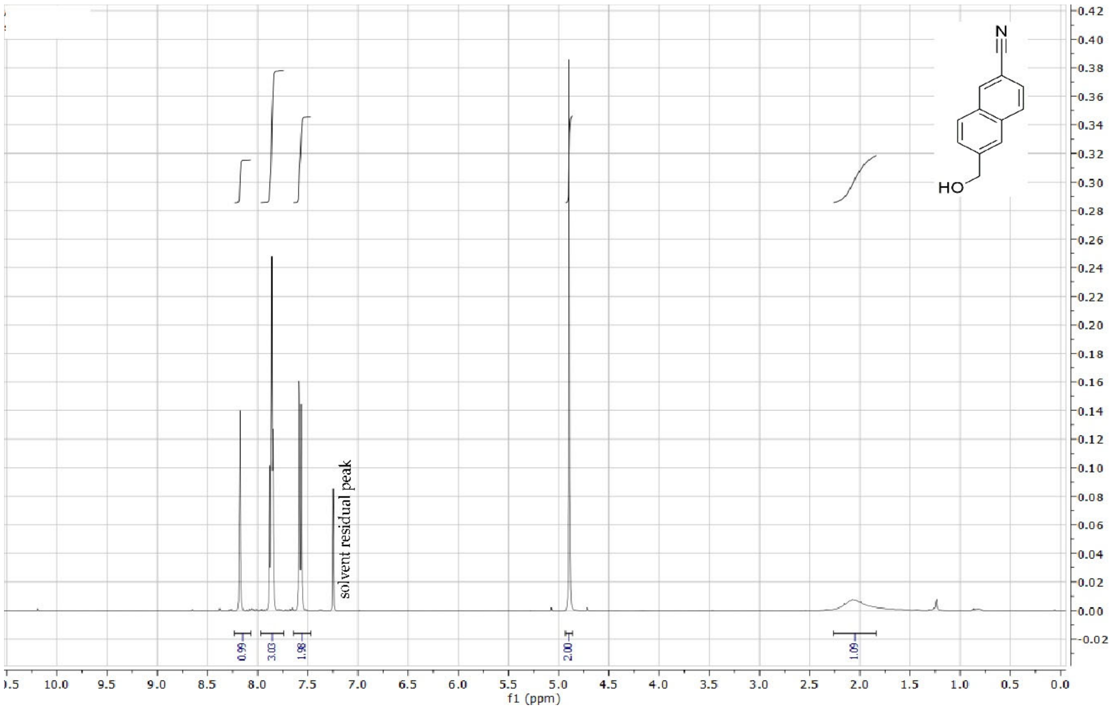

Figure S6. ^{1}H NMR for the 6-(Hydroxymethyl)-2-naphthonitrile (**5**).

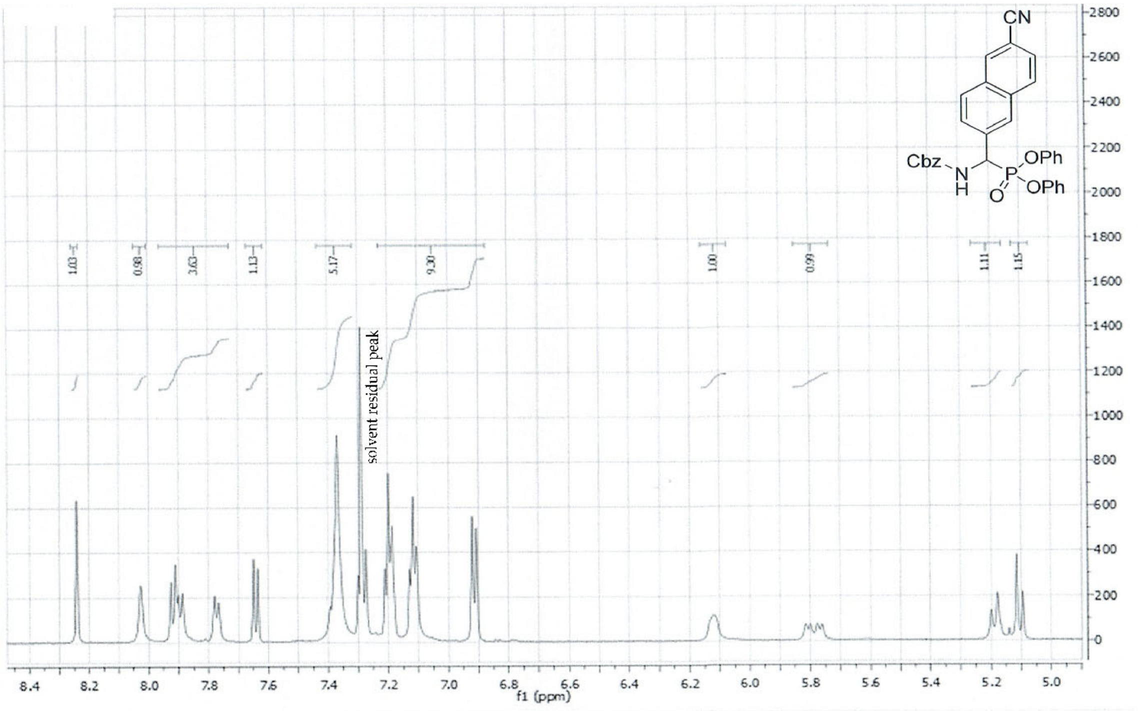

Figure S7. ^{1}H NMR for the Cbz-6-CN-NphthP(OPh)$_2$ (**7**).

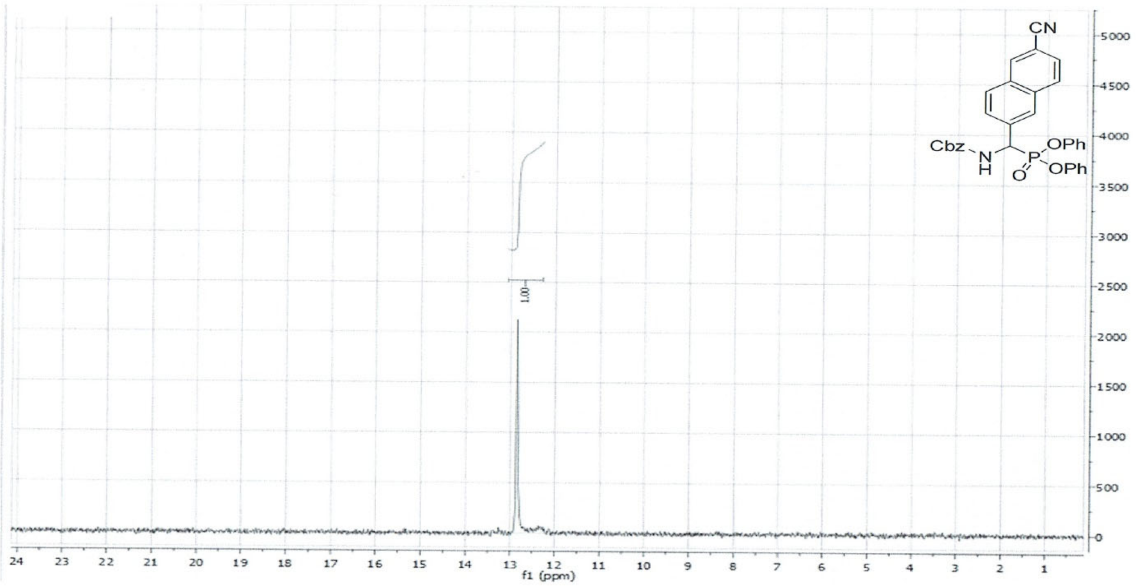

Figure S8. ^{31}P NMR for the Cbz-6-CN-NphthP(OPh)2 (**7**).

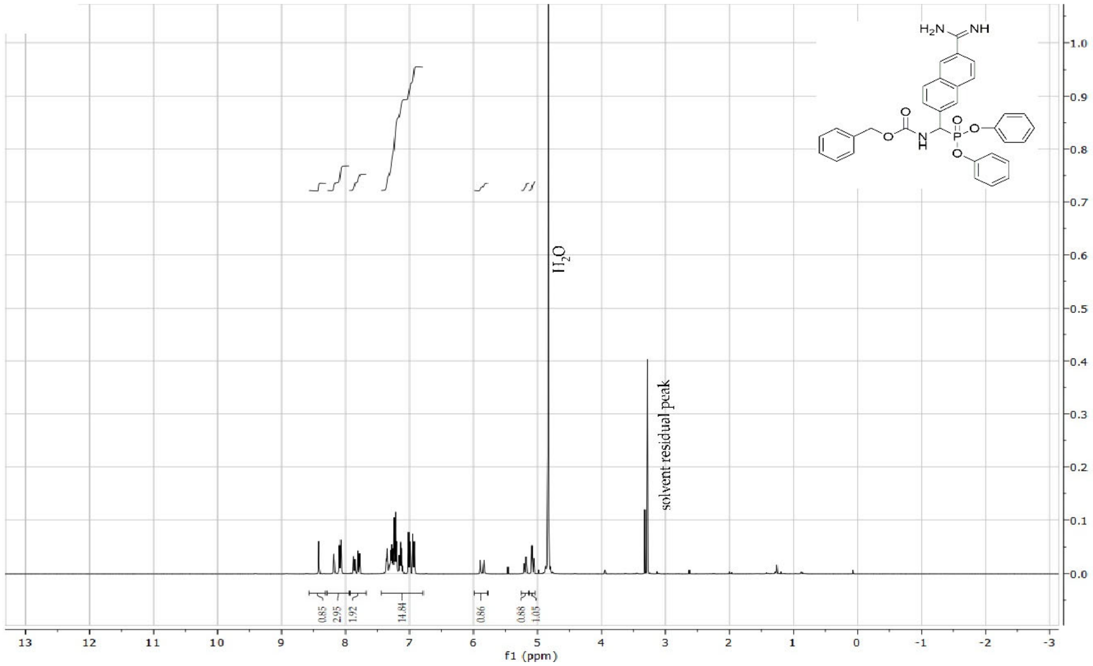

Figure S9. ^{1}H NMR for the Cbz-6-AmNphth^{P}(OPh)2 (**8**).

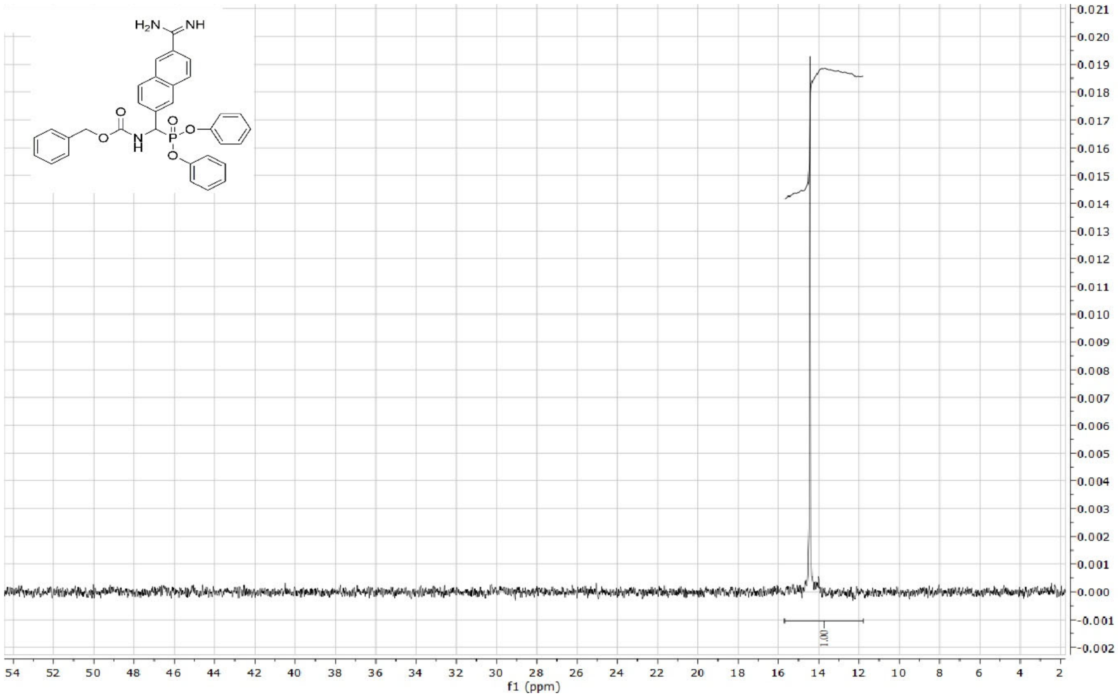

Figure S10. ^{31}P NMR for the Cbz-6-AmNphth^{P}(OPh)$_2$ (**8**).

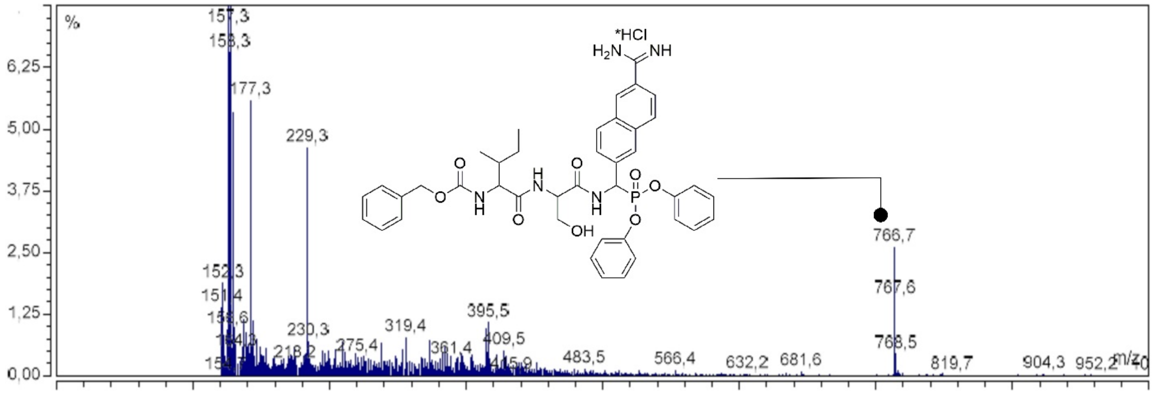

Figure S11. MS analysis for Cbz-Ile-Ser-6-AmNphth^{P}(OPh)$_2$ (**11**).

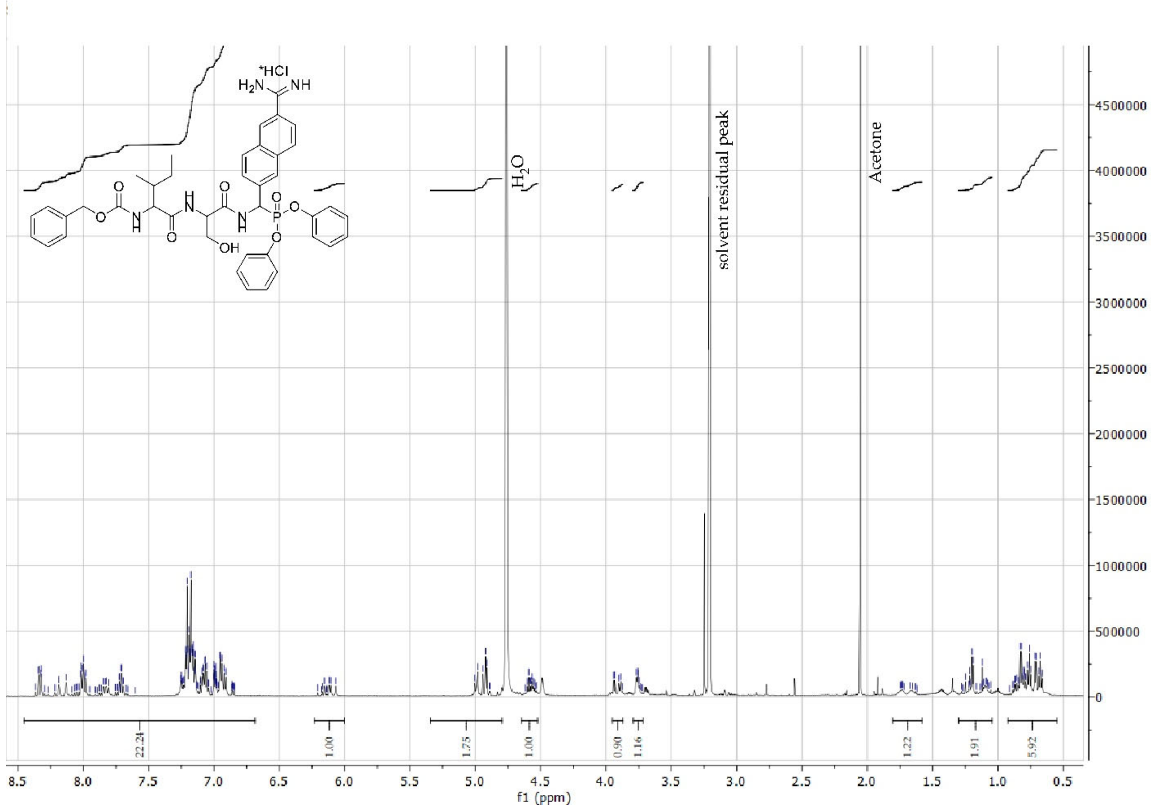

Figure S12. ^{1}H NMR for Cbz-Ile-Ser-6-AmNphth^{P}(OPh)$_2$ (**11**).

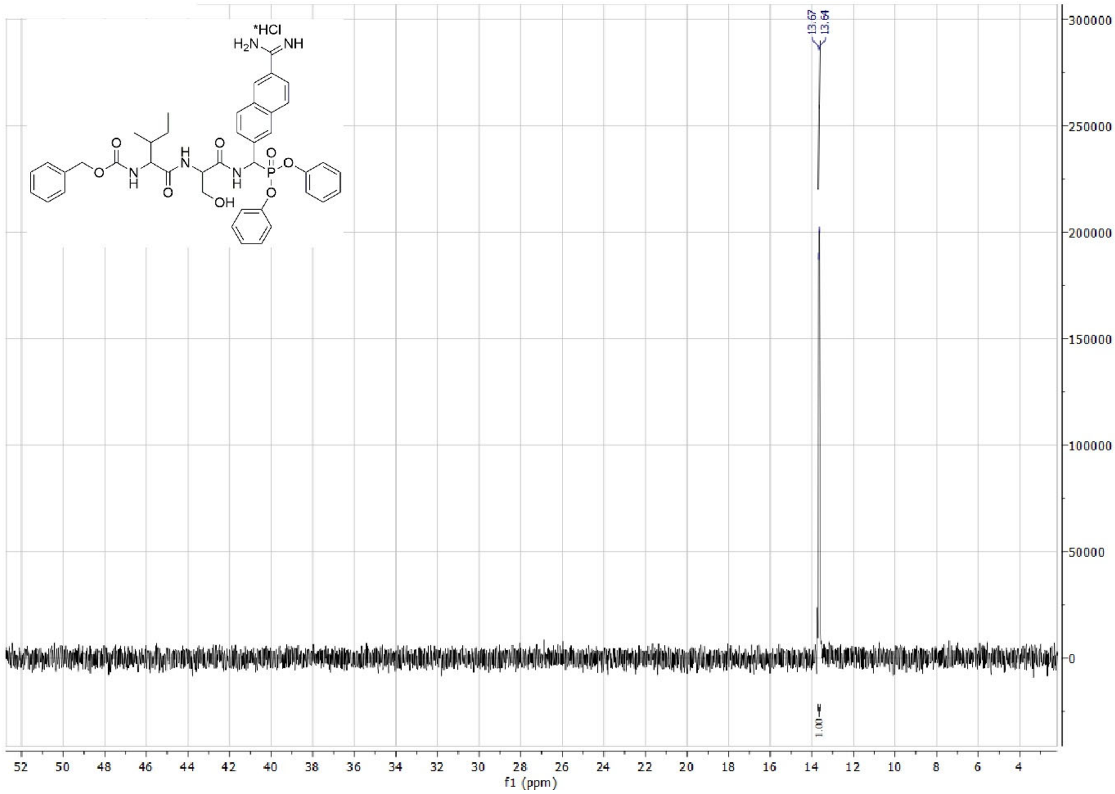

Figure S13. ^{31}P NMR for Cbz-Ile-Ser-6-AmNphth^{P}(OPh)$_2$ (**11**).
